# Supplementary figures and images for: A versatile mouse model to advance human microglia transplantation research in neurodegenerative diseases
Source: Mol Neurodegener. 2025 Mar 11;20:29. doi: 10.1186/s13024-025-00823-2 (PMC11895352; doi:10.1186/s13024-025-00823-2)

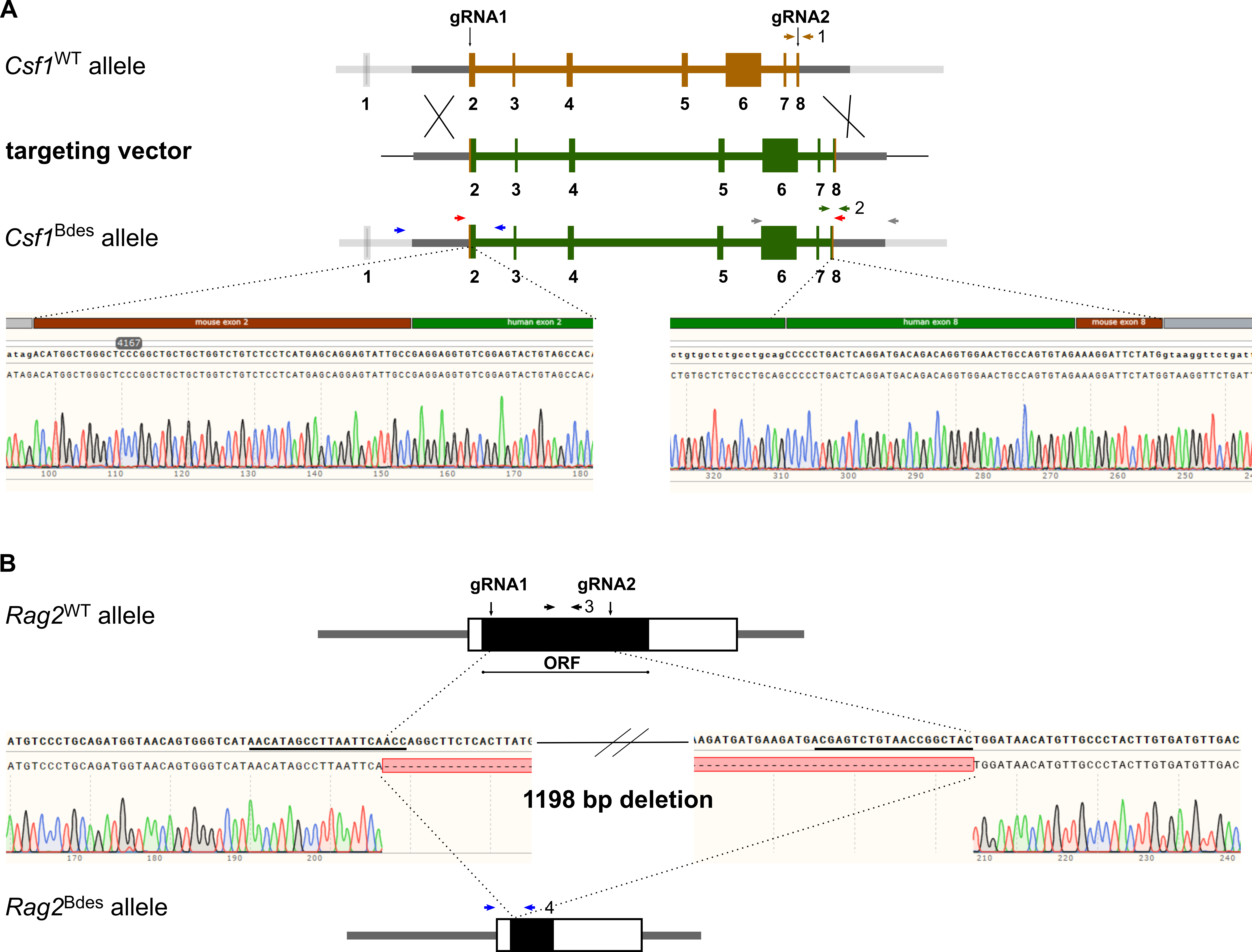

Supplement: Supplementary file 1 — Additional file 1. Generation of the humanized Csf1 and Rag2 knock mice. A. Schematic overview of the strategy used to generate the humanized Csf1KI allele. Exons are presented as boxes; The positions of the guide RNA’s are indicated. Primers used for quality control by PCR and Sanger sequencing are depicted as arrows. The mouse exons and introns presented in brown are replaced by human exons and introns indicated in green. The lower panel shows the Sanger sequencing results at the recombination sites. Genotyping primers are indicated with brown arrows (1) for theCsf1WT allele and green (2) for the Csf1Bdes allele. B. Schematic overview of the strategy used to generate the Rag2Bdes KO allele. Exon3 of the mouse Rag2 gene is depicted as a box, the complete open reading frame of the Rag2 gene as a black box, the position of the two guide RNA’s are indicated. The lower part shows the Sanger sequencing results at the deletion site demonstrating that 1198 bp of the coding sequence are deleted. The primers used to genotype the alleles are indicated with arrows, black arrows (3) for Rag2WTallele and blue arrows (4) for Rag2Bdesallele. [file 13024_2025_823_MOESM1_ESM.png]

## Slide 1
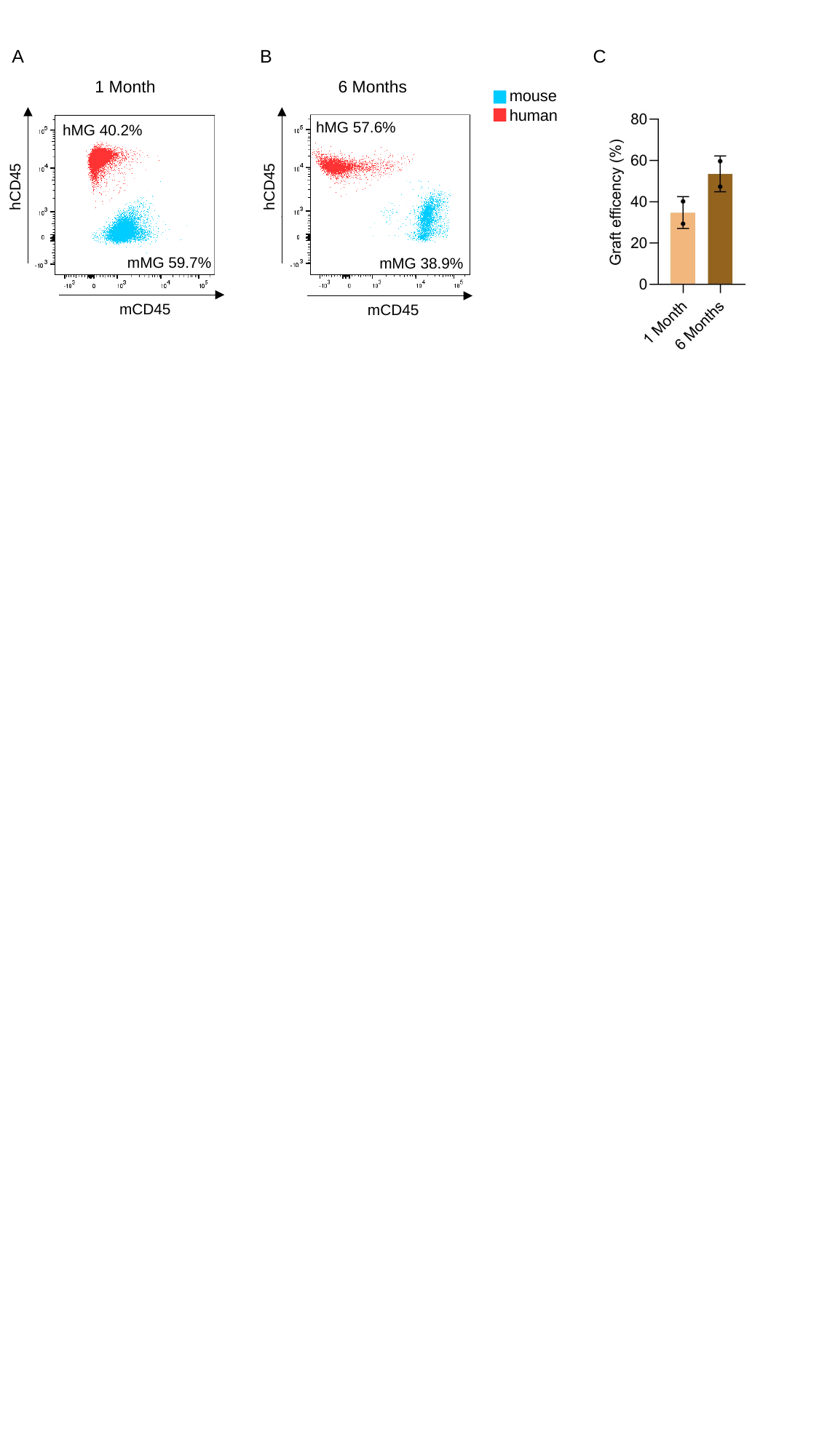

A
B
C
1 Month
6 Months
mouse
human
hMG 57.6%
hMG 40.2%
hCD45
hCD45
mMG 59.7%
mMG 38.9%
mCD45
mCD45

Supplement: Supplementary file 2 — Additional file 2. Efficient xenotransplantation of human derived microglia in the hCSF1Bdesmouse model. A-B.hCSF1Bdes mice were xenografted with human microglia derived from iPSCs (UKBi011-A-3). Isolated microglia were analyzed by flow cytometry one month (A) and 6 months (B) after xenotransplantation. Human microglia (hMG) and mouse microglia (mMG) are represented as percentage of total CD11b+ cells. C. Graft efficiency of hMG at 1 and 6 months after transplantation. n = 2, bar plot represents mean ± SD. [file 13024_2025_823_MOESM2_ESM.pptx]

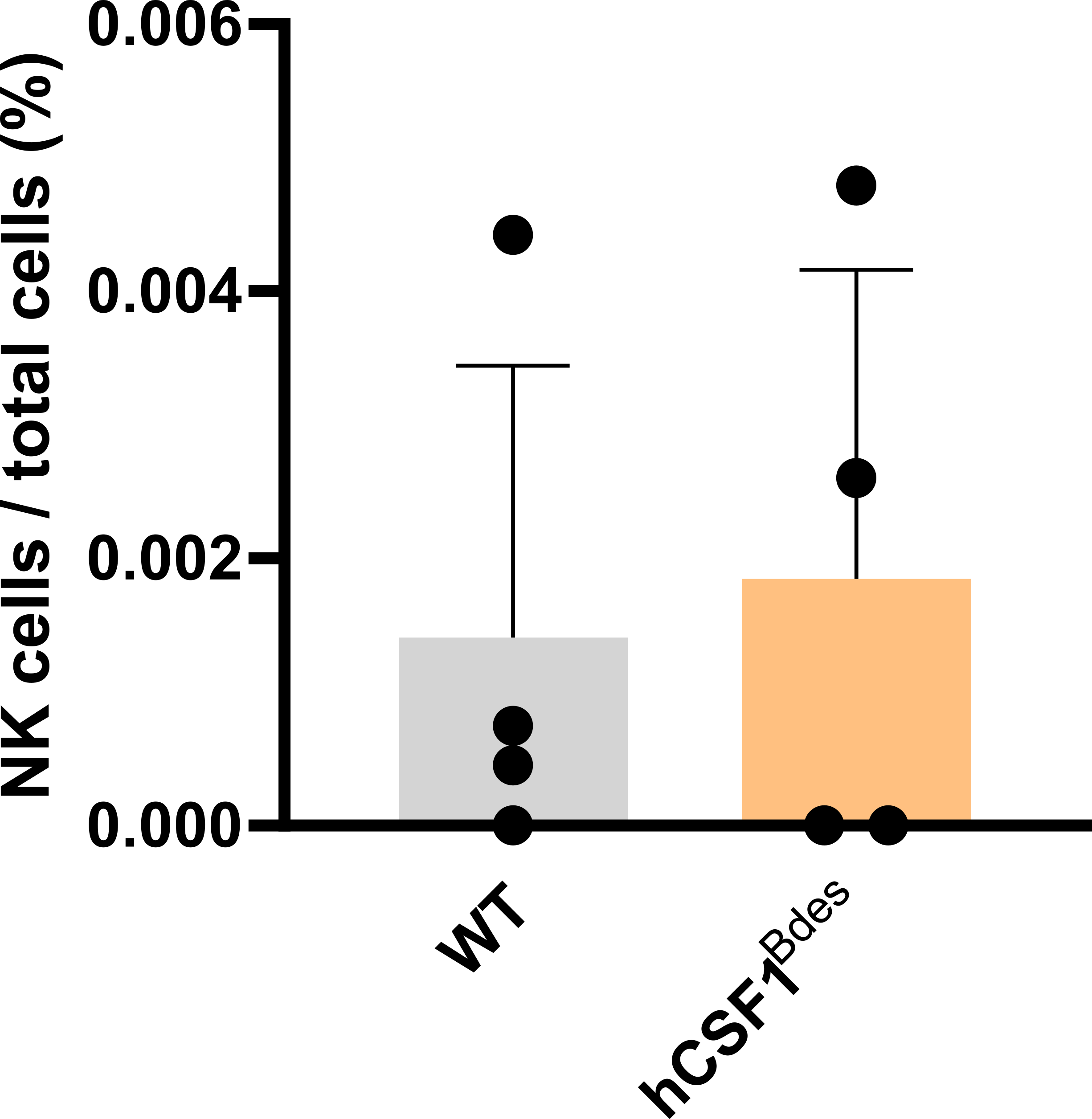

Supplement: Supplementary file 3 — Additional file 3. Single cells have been isolated from one hemisphere of a mouse brain and analysed by FACS. The graph represents FACS quantification of NK cell number in hCSF1Bdes and in WT mice. We express the number of NK cells, as % of cells of the total isolated mouse cells. The total number of cells we obtain from one mouse is variable, averaging ± 500.000 cells/mouse hemibrain (omitting cerebellum). Bar plot represents mean ± SD (n = 4, Mann Whitney U test). [file 13024_2025_823_MOESM3_ESM.png]
